# Supplementary material for: Identification of photoperiod-induced specific miRNAs in the adrenal glands of Sunite sheep (Ovis aries)
Source: Front Vet Sci. 2022 Jul 22;9:888207. doi: 10.3389/fvets.2022.888207 (PMC9354845; doi:10.3389/fvets.2022.888207)
Supplement: Supplementary file 1 [file Data_Sheet_1.ZIP › Supplementary file/miRDeep2 file.zip!/miRDeep2 file/Know_Structure/SPLP42A2/oar-mir-125b.pdf]

miRBase precursor : oar-mir-125b
Total read count : 222128
oar-miR-125b read count : 221494
remaining reads : 634

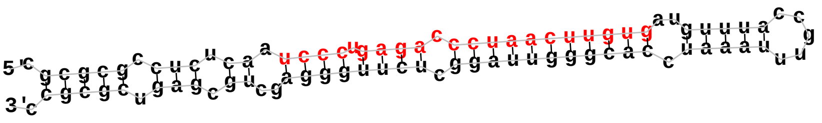

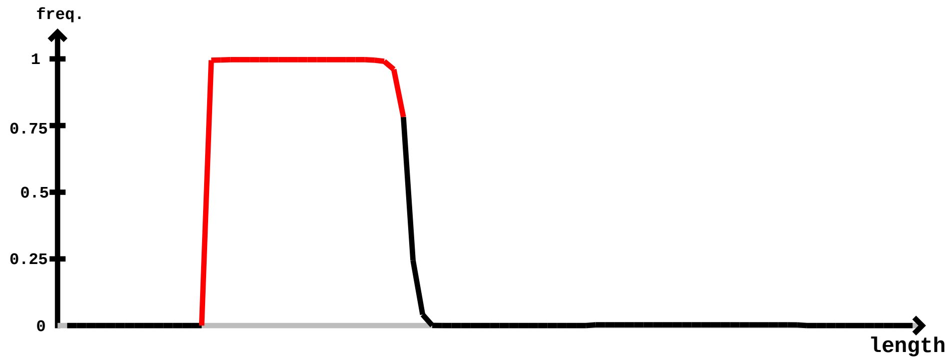

oar-miR-125b

5'- cgcgcgccucucaaucccugagacccuaacuugugauguuuaccguuuaaauccacggguuaggcucuugggagcugcgagucgcgcc -3'	exp		
.(((((.(((.((.((((.((((.((((((((((..(((((.....))))).)))))))))).))))))))..)).))).))))).	reads	mm	sample
............aaucccugagacccuaacuugug...	1	0	seq
.............aucccugagacccuaacuugu..	3	0	seq
.............aucccugagacccuaacuugug...	2	0	seq
..............ucccugagGcccuaacu...	1	1	seq
..............ucccugagacccuGacu...	1	1	seq
..............ucccAgagacccuaacu...	2	1	seq
..............ucccugagacccAaacu...	1	1	seq
..............ucccugagaUccuaacu...	2	1	seq
..............ucccugagacccuaacC...	61	1	seq
..............Acccugagacccuaacu...	5	1	seq
..............ucccugagacccuaCcu...	2	1	seq
..............ucccugagacccuaacu...	381	0	seq
..............ucccugagacccuaacuu..	926	0	seq
..............ucccugagacccuaacCu..	14	1	seq
..............ucccuAagacccuaacuu..	1	1	seq
..............ucccugagacccuaacuug...	6708	0	seq
..............ucccugagacccuaacuugu..	39656	0	seq
..............ucccugagacccuaacuugug...	119196	0	seq
..............ucccugagacccuaacuuguga..	44932	0	seq
..............ucccugagacccuaacuugugau...	4975	0	seq
..............ucccugagacccuaacuugugaG...	4037	1	seq
..............ucccugagacccuaacuugugaGg..	42	1	seq
..............ucccugagacccuaacuugugaug..	34	0	seq
..............ucccugagacccuaacuugugaugu...	29	0	seq
...............cccugagacccuaacuu..	1	0	seq
...............cccugagacccuaacuug...	5	0	seq
...............cccugagacccuaacuugu..	14	0	seq
...............cccugagacccuaacuugug...	28	0	seq
...............cccugagacccuaacuuguga..	151	0	seq
...............cccugagacccuaacuugugaG...	8	1	seq
...............cccugagacccuaacuugugau...	5	0	seq
...............cccugagacccuaacuugugaGg..	1	1	seq
................ccugagacccuaacuug...	13	0	seq
................ccugagacccuaacuugu..	45	0	seq
................ccugagacccuaacuugug...	145	0	seq

oar-miR-125b

```
cgcgcgccucucaaucccugagacccuaacuugugauguuuaccguuuaaauccacggguuaggcucuugggagcugcgagucgcgcc
................ccugagacccuaacuuguga....................................................   47   0   seq
................ccugagacccuaacuugugau...................................................   4    0   seq
................ccugagacccuaacuugugaG...................................................   5    1   seq
.................cugagacccuaacuugug.....................................................   3    0   seq
.................cugagacccuaacuuguga....................................................   3    0   seq
.................cugagacccuaacuugugaGgu.................................................   1    1   seq
..................ugagacccuaacuugug.....................................................   3    0   seq
...........................................cguuuaaauccacggguuaggcucuugggagcu............   1    0   seq
................................................aaauccacggguuaggcucuu...................   1    0   seq
.....................................................cacggguuaggcucuugggagcu............   1    0   seq
......................................................acggguuaggcucuuggg................   1    0   seq
......................................................acggguuaggcucuugggag..............   9    0   seq
......................................................acggguuaggcucuugggagc.............   78   0   seq
......................................................acggguuaggcucuugggagcu............   533  0   seq
......................................................acggguuaggcucuugggagcug...........   9    0   seq
......................................................acggguuaggcucuugggagcugc..........   1    0   seq
```
